# Editorial: Higher Education Dropout After COVID-19: New Strategies to Optimize Success

**DOI:** 10.3389/fpsyg.2022.880295

**Published:** 2022-04-08

**Authors:** Ana B. Bernardo, Adrian Castro-Lopez, Alejandro Diaz Mujica

**Affiliations:** ^1^Department of Psychology, University of Oviedo, Oviedo, Spain; ^2^Department of Business Administration, University of Oviedo, Oviedo, Spain; ^3^Department of Psychology, University of Concepcion, Concepción, Chile

**Keywords:** higher education, drop-out, self-regulation, educational quality, hybrid learning

Over the last few years, several challenges have been identified in higher education. On the one hand, a high percentage of students drop out of university in the first year. This problem has been exacerbated in the context of the deregulation of teaching and learning conditions in physical distance due to the COVID-19 pandemic. On the other hand, during the years of the pandemic, educational institutions, under pressure to adapt their teaching procedures, have made significant efforts to increase the integration of technology in teaching and to update the skills of their teachers in the use of virtual teaching resources.

The improvement of teaching and learning processes requires a proactive behavior by lecturers and students. The need for scientific knowledge that supports teaching procedures that involve the intentional promotion of various cognitive-motivational variables that influence learning is clearly visible. In these efforts, it is possible to find support in the persistent appearance of new technological resources that improve communications, access, and availability of knowledge.

Lecturers play a fundamental role by embodying models of behaviors and verbalizations of thoughts during the teaching of their subjects and in the various interactions with their students. In addition, academic performance is related to the adequate use of skills to develop learning processes in university studies. In both aspects, in the last decades, research has provided remarkable developments in the knowledge of cognitive, social, and motivational variables that are at the basis of academic performance.

However, in the context of the pandemic, psychology, and education, lecturers from different academic disciplines have been particularly challenged to answer questions that encompass all multi-causal variables (affective, cognitive, and social) that influence dropout, across different levels of analysis, including: (1) Do we have the necessary measuring instruments for variables involved in teaching and learning processes? What are the relationships between these variables? How adequate are the hypothesized relationship models? (2) What is the degree of impact of the intentional modification programs of these variables? What are their conceptual foundations? What are the measures of their impacts? And (3) if these programs are successful, how to evaluate their application to large numbers of participants?

In order to contribute to answering these questions, this special issue provides interesting systematizations and reflections on innovations, proposed solutions, and experiences obtained by students and teachers in the context of the COVID-19 pandemic. The research presented here is characterized by offering rigorous, clear, replicable views and procedures, which constitute valuable contributions with a general constructive vision in the advancement of scientific knowledge. They can guide study and lecturing practices and offer theoretical foundations for the design of university policies to reduce dropout rates and improve lecturing and learning processes.

## Author Contributions

All authors listed have made a substantial, direct, and intellectual contribution to the work and approved it for publication.

## Conflict of Interest

The authors declare that the research was conducted in the absence of any commercial or financial relationships that could be construed as a potential conflict of interest.

## Publisher's Note

All claims expressed in this article are solely those of the authors and do not necessarily represent those of their affiliated organizations, or those of the publisher, the editors and the reviewers. Any product that may be evaluated in this article, or claim that may be made by its manufacturer, is not guaranteed or endorsed by the publisher.

